# Hossain MS, et al. Human Health Risk of Chromium Intake From Consumption of Poultry Meat and Eggs in Dhaka, Bangladesh. *J Health Pollution*. 2017;7(14):30–36.

**DOI:** 10.5696/2156-9614-7.16.1

**Published:** 2017-12-18

**Authors:** Jack Caravanos

**Affiliations:** Clinical Professor of Environmental Public Health Sciences, College of Global Public Health, New York University, jack@pureearth.org

The following article: Md. Shakhaoat Hossain, Prantik Roy, Monira Islam, Alamgir Zaman Chowdhury, Zeenath Fardous, Md. Abdur Rahman, ASM Salfullah, Mahmudul Hasan, Md. Mazibur Rahman. Human Health Risk of Chromium Intake From Consumption of Poultry Meat and Eggs in Dhaka, Bangladesh, https://doi.org/10.5696/2156-9614-7.14.30, published in the Journal of Health and Pollution, Volume 7, Edition 14 (June 2017) pp. 30–36 has been retracted at the request of the authors due to inadvertent cross contamination of samples in the laboratory. The error was discovered after publication.

The authors regret any inconvenience to the scientific community.

